# Machine Learning-Based Predictive Models of Behavioral and Psychological Symptoms of Dementia

**DOI:** 10.1093/geroni/igab046.2446

**Published:** 2021-12-17

**Authors:** Eunhee Cho, Sujin Kim, Seok-Jae Heo, Jinhee Shin, Byoung Seok Ye, Jun Hong Lee, Bada Kang

**Affiliations:** 1 Yonsei University College of Nursing, Seoul, Seoul-t'ukpyolsi, Republic of Korea; 2 Yonsei University, Seoul, Seoul-t'ukpyolsi, Republic of Korea; 3 Yonsei University Graduate School, Seoul, Seoul-t'ukpyolsi, Republic of Korea; 4 National health insurance service Ilsan hospital, Goyang, Kyonggi-do, Republic of Korea; 5 Mo-Im Kim Nursing Research Institute, Yonsei University College of Nursing, Seoul, Seoul-t'ukpyolsi, Republic of Korea

## Abstract

Models predicting the occurrence of specific types of behavioral and psychological symptoms of dementia (BPSD) can be highly beneficial for its early intervention and individualized care planning. Using a machine learning approach, this study developed and validated predictive models of the occurrence of BPSD, categorized into seven subsyndromes, among community-dwelling older adults with dementia in South Korea. BPSD dairy was used to measure BPSD and the state of unmet needs daily. We measured sleep and activity levels using actigraphy, and stress and fatigue using a portable heart rate variability analyzer. We developed predictive models and conducted cross-validation using training data that consisted of the first two wave dataset, and then validated the models using wave 3 test data. To deal with imbalanced datasets, we used Synthetic Minority Oversampling Technique (SMOTE), an over-sampling method. Categorical variables were pre-processed using target encoding. We then compared the machine-learning models with logistic regression. The area under the receiver operating characteristic curve (AUC) scores of the support vector machine (SVM) models for the wave 3 test data showed a similar or greater value than logistic regression models across all BPSD subsyndromes. The SVM model (AUC = 0.899) had an AUC value greater than that of the logistic regression model (AUC = 0.717), particularly for hyperactivity symptoms. Machine learning algorithms, especially SVM models, can be used to develop BPSD prediction models to help identify at-risk individuals and implement symptom-targeted individualized interventions.

